# Editorial: Ketamine for treatment of acute and chronic pain: The relationship of mechanism and exposure to therapeutic outcome

**DOI:** 10.3389/fpain.2022.991569

**Published:** 2022-08-23

**Authors:** Albert Dahan, Thomas K. Henthorn

**Affiliations:** ^1^Department of Anesthesiology, Leiden University Medical Center, Leiden, Netherlands; ^2^PainLess Foundation, Leiden, Netherlands; ^3^Department of Anesthesiology, University of Colorado School of Medicine, Aurora, CO, United States; ^4^Department of Pharmaceutical Sciences, Skaggs School of Pharmacy and Pharmaceutical Sciences, University of Colorado, Aurora, CO, United States

**Keywords:** ketamine, *S*-ketamine, ketamine analogs, mood disorder, sublingual ketamine

Ketamine is by far one of the more intriguing drugs used in anesthesia, pain management, and in recent years psychiatry. Ketamine has been with us since the early 1960's and is used clinically for various indications that include induction and maintenance of anesthesia, sedation, treatment of acute and chronic pain, management of therapy-resistant depression, and other psychiatric illnesses such as posttraumatic stress disorder. Interestingly, its efficacy for each of these indications is dose-dependent with anesthesia requiring the highest dose, followed by pain relief at lower doses, and antidepression requiring the lowest dose. Ketamine is certainly not a simple or straightforward drug; it has two active stereoisomers, *S-* and *R-*ketamine, and multiple sequential metabolites including norketamine, hydroxynorketamine, and dehydronorketamine, all with varying pharmacological activity (1, 2). Importantly, apart from producing its dose-dependent desired effects, ketamine produces unwanted psychedelic effects that limit dosing recommendations as well as patient compliance. Ketamine is within the class of drugs termed psychoplastogens, a group of drugs that promote synaptic cross-talk in the cortex through rewiring of neurocircuitry (3, 4). Ketamine and the other psychoplastogens produce mind-altering effects that are highly associated with their desired effects (5, 6). As more evidence emerges, it is clear that many aspects of the pharmacology of ketamine, both with respect to its pharmacokinetics and pharmacodynamics, are still poorly understood and require further study.

*Frontiers in Pain Research* hosted the Research Topic “*Ketamine for treatment of acute and chronic pain: the relationship of mechanism and exposure to therapeutic outcome*” to improve our understanding of ketamine therapy and to share various new developments in the field. This led to the publication of four papers of interest (Willis and Goldstein; Voss et al.; Simons et al., a; Simons et al., b). The first paper by Willis and Goldstein discusses the ability of short infusions of ketamine to prevent the transition of acute to chronic postsurgical pain, possibly through non-*N*-methyl-D-aspartate (non-NMDA) receptor systems, most importantly through HCN1 (hyperpolarization-activated cyclic nucleotide-regulated type 1) channels. In this highly interesting and informative paper, the authors discuss, amongst other things, the risk factors for development of persistent postsurgical pain. One important risk factor relates to the presence of specific psychological traits such as anxiety, depression and pain catastrophizing. Possibly ketamine will reduce chronic pain development through improvement of such imprinted behavior. However, as the authors hypothesize, patients need to be aware of the effects of ketamine: “… awareness of the dissociated state is necessary for the antidepressant effects of ketamine and it is those effects which are critical in preventing the development of chronic postsurgical pain …” This is an intriguing hypothesis and suggests that ketamine should be given to patients prior to the induction of anesthesia (in this respect timing of administration is essential), and equally important, that we should be careful titrating the patient's ketamine experience. In agreement with this suggestion, short-term ketamine analgesia is similarly dependent on the experience that ketamine induces (5, 6). As the authors correctly state, ketamine might not work for all patients, and identification of patients that will respond is warranted and deserves further study.

In the next paper, Voss et al. discuss non-NMDA analgesia of ester-analogs of ketamine. Such analogs are rapidly metabolized in plasma and, as the authors state, are being developed to circumvent ketamine's hallucinogenic effects. Whether this will be the case when used clinically deserves further study as it might be necessary to administer such short-acting compounds by continuous infusion (*cf*. remifentanil and remimazolam). In their paper, the authors summarize recent research on the ketamine-analog R5, which is an ester with almost 200-times less affinity for the NMDA receptor compared to ketamine. Still R5 has a similar analgesic potency as ketamine which indicates that ketamine's analgesic properties are (partly) independent of the NMDA receptor. Indeed, ketamine has been shown to produce part of its analgesic effects through activation of other receptor systems including the mu-opioid receptor and the β-common receptor CD131 (7, 8). R5, as well as ketamine, acts at a specific potassium channel (TWIK K2P) known to modulate pain. The authors further discuss the anatomic location of the antinociceptive effects of R5 and suggest the limbic system as an important site of action, thereby diminishing the emotional coloring of pain rather than by reducing the afferent nociceptive input to the brain as opioids do.

Finally, in two connected papers, Simons et al., a; Simons et al., b present pharmacokinetic and pharmacodynamic modeling data of an *S*-ketamine oral thin film (SK-OTF) for sublingual or oral placement. The studies were performed in young, healthy volunteers of either sex. The model describes the pharmacokinetics of the parent drug and its two sequential metabolites (norketamine and hydroxynorketamine), and describes the direct uptake of *S-*ketamine through the oral mucosa as well as the uptake in the gut following ingestion of the SK-OTF. Various locations of drug metabolism are discussed: mucosa, enterocytes and/or liver. The SK-OTF has high first-pass metabolism with formation of large amounts of norketamine and hydroxynorketamine with bioavailability <30%; in comparison bioavailability is <24% after oral ketamine consumption, around 50% after intranasal administration and ~70% with inhalation of ketamine areosols. In their second paper, Simons et al., b model the pharmacokinetic-pharmacodynamic profile of the SK-OTF and describe the rapid-onset and long-lasting antinociceptive efficacy of the SK-OTF in three experimental nociceptive assays without any contribution from either norketamine or hydroxynorketamine. This suggests that this formulation may be used for various conditions, including postoperative pain, breakthrough pain and in all conditions in which high concentrations of particularly hydroxynorketamine in particular are deemed necessary (e.g., therapy-resistant depression and posttraumatic stress disorder).

The four studies in this Research Topic each improve our current knowledge and propose novel strategies for the application of ketamine and its analogs. Ketamine and its metabolites will certainly be the Research Topic of many more relevant studies in the near future. Finally, we are all aware of the ongoing opioid pandemic that is partly related to abuse of illicit, mostly synthetic opioids (predominantly in the USA and Canada) and partly related to relentless prescription of licit opioids by physicians across a range of specialties (predominantly in the EU) (9, 10). This Research Topic of articles in *Frontiers in Pain Research* strongly suggest that the wise and targeted use of ketamine in the treatment of acute pain and in the prevention of chronic pain is a serious alternative to opioids and, consequently, could significantly reduce the damage that opioids do to individual patients, their family and friends, and society as a whole.

## Author contributions

All authors listed have made a substantial, direct, and intellectual contribution to the work and approved it for publication.

## Conflict of interest

The authors declare that the research was conducted in the absence of any commercial or financial relationships that could be construed as a potential conflict of interest.

## Publisher's note

All claims expressed in this article are solely those of the authors and do not necessarily represent those of their affiliated organizations, or those of the publisher, the editors and the reviewers. Any product that may be evaluated in this article, or claim that may be made by its manufacturer, is not guaranteed or endorsed by the publisher.
